# Three dimensional structure prediction and proton nuclear magnetic resonance analysis of toxic pesticides in human blood plasma

**DOI:** 10.7555/JBR.26.20110132

**Published:** 2012-04-15

**Authors:** Amit Kumar Sharma, Rajeev Kumar Tiwari, Mulayam Singh Gaur

**Affiliations:** Pesticides Research and Sensors Laboratory, Department of Physics, Hindustan College of Science and Technology, Farah, Mathura-281122 (U.P.), India.

**Keywords:** ^1^H-NMR, toxic pesticide, nuclear magnetic resonance (NMR) parameter, relaxation time, computational tool, data interpretation

## Abstract

The purpose of this study was to investigate the nuclear magnetic resonance (NMR) assignments of hydrolyzed products extracted from human blood plasma. The correlations between chemical, functional and structural properties of highly toxic pesticides were investigated using the PreADME analysis. We observed that toxic pesticides possessed higher molecular weight and, more hydrogen bond donors and acceptors when compared with less toxic pesticides. The occurrence of functional groups and structural properties was analyzed using ^1^H-NMR. The ^1^H-NMR spectra of the phosphomethoxy class of pesticides were characterized by methyl resonances at 3.7-3.9 ppm (*δ*) with the coupling constants of 11-16 Hz (*J_P-CH3_*). In phosphoethoxy pesticides, the methyl resonance was about 1.4 ppm (*δ*) with the coupling constant of 10 Hz (*J_P-CH2_*) and the methylene resonances was 4.2-4.4 ppm (*δ*) with the coupling constant of 0.8 Hz (*J_P-CH3_*), respectively. Our study shows that the values of four parameters such as chemical shift, coupling constant, integration and relaxation time correlated with the concentration of toxic pesticides, and can be used to characterise the proton groups in the molecular structures of toxic pesticides.

## INTRODUCTION

Pesticides cause disease or damage to an exposed organism when used by farmers in agricultural fields or domestic applications[1]. It is of great interest to predict and characterize the ^1^H nuclear magnetic resonance (NMR) spectra of pesticides in biological mechanisms. The NMR parameters are closely linked with the molecular structures of highly toxic pesticides[2]-[3]. Chemical shifts of proton nuclei in the molecular structure describe the bonding and electronic effects associated with ^1^H-NMR spectra of highly toxic pesticides. It also characterizes spin-spin coupling that explains the position of neighboring nuclei in different molecular structures of highly toxic pesticides[2],[3]. Over the last two decades, increased utilization of enabling methodology such as Fourier transform infrared spectroscopy (FTIR), ultraviolet-visible spectrophotometry (UV-Vis) and flow chemistry has led to an increases in the number of compounds[4],[5]. This has led to a significant increase in the quantity of analytical data that needs to be reviewed to characterize the toxic pesticides[6]-[10]. Each NMR parameter may play an important role in determining the structure of pesticides. Detailed study of pesticides is required so as to determine which compounds present long-term residual effects as opposed to those which are readily biodegradable or undergoing photochemical degradation[11],[12].

Moreover, the degradation and harmful effects of the metabolic products of pesticides remain unknown. Obviously, NMR can be effectively used for the determination of the structure of such toxic pesticides under various environments[13]-[16]. Generally, there are four types of information obtained from NMR spectrum including chemical shift, integrated intensity, relaxation time and coupling constant. Each of these data provides unique information for use in the determination of the structure of pesticides. The chemical shift value allows investigators to determine the types of protons present in samples, i.e., protons bonded to saturated carbons, olefinic compounds, aromatic carbon, heteroatom or carbon with functional groups like aldehydes (-CHO). In addition, it can be further used to distinguish protons with specific types such as methyl (-CH_3_), methylene (CH_2_) and methine (-CH) protons. The organophosphate pesticides mainly consist of phosphate or thiophosphate esters with the general formula: 
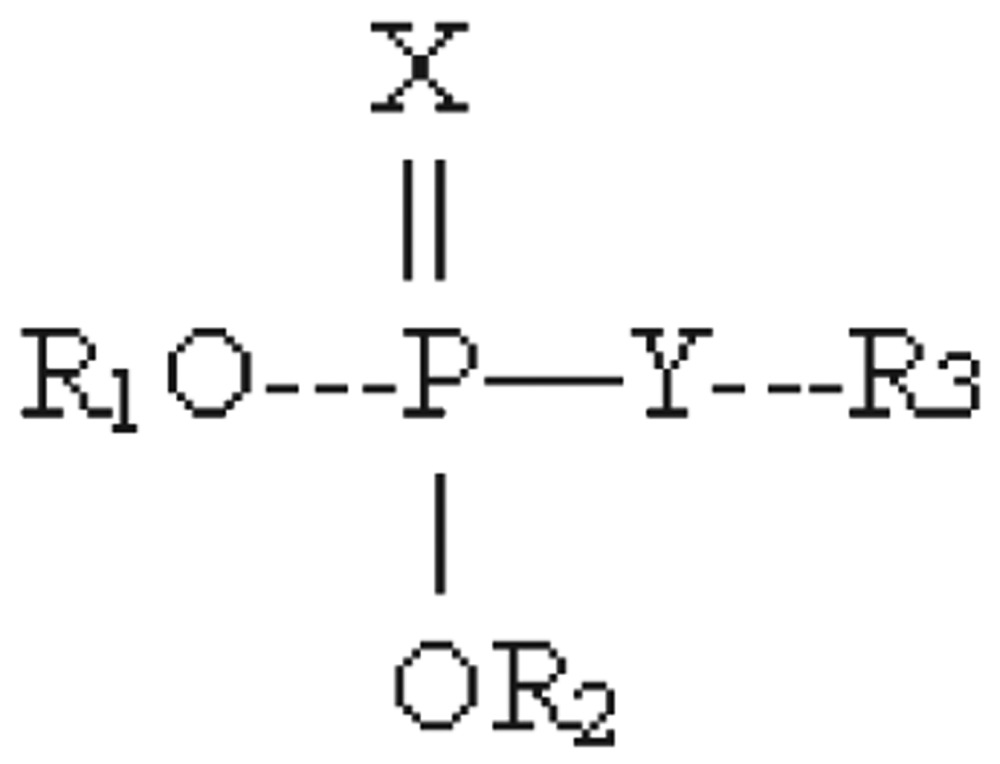


Where R_1_ and R_2_ are mostly methyl or ethyl groups, and R_3_ is a large organic group. In these compounds, X is oxygen or sulfur and Y is oxygen, sulfur or nitrogen. The ^1^H-NMR spectra of the phosphomethoxy class of compounds are characterized by methyl resonances at 3.7-3.9 ppm (*δ*) with the coupling constant of 11-16 Hz (*J_P-CH3_*), and in phosphoethoxy class, the methyl resonance is obtained at 1.4 ppm (*δ*) with coupling constant of 10 Hz (*J_P-CH2_*), and 4.2-4.4 ppm (*δ*) with the coupling constant of 0.5-0.8 Hz (*J_P-CH3_*), respectively. With various substituent groups at R_3_, interpretation of spectra may require considerable effort[17]-[22].

The chemical, structural and physiochemical properties of toxic pesticides were investigated using ^1^H-NMR analysis. The objective of ^1^H-NMR analysis is to detect highly toxic pesticides at different concentrations through the proposed process and to explore the effective parameters. These parameters are useful to predict the toxic pesticides and they can be applied as a detection tool. As this is new information, there was no evidence regarding the integration ratio and relaxation time for these toxic pesticides until this study, which provides the quantitative information regarding toxic pesticides

## MATERIALS AND METHODS

### Apparatus

^1^H-NMR spectrometer (Bruker, 300 MHz and 500 MHz) was used in methanol- d_4_/CdCl_3_ solvents for multi-residue analysis of environmental pollutants. An ultrasound sonicator (Remi, India) was used to homogenize toxic pesticides present in blood samples. The instrument parameters were adjusted according to the installation manual.

### Reagents and sample preparation

Methyl parathion (98%), parathion (97%), malathion (96%) sodium hydroxide, acetone, double distilled water, methanol, hydroxyl ammonium hydrochloride and acetonitrile were purchased from Merck Chemicals International (Darmstadt, Germany). Chemicals were high-performance liquid chromatography (HPLC) grade.

Plasma samples were collected from subjects aged 25-35 years and placed in 5 mL vacutaineer lithium heparin glass tube. The samples were then centrifuged at 2500 rpm for 20 min and the supernatant was stored in a 5 mL glass vial at -20°C. After separation of human plasma samples, the known toxic pesticides were mixed and extracted three times using the liquid-liquid extraction method with dichloromethane (DCM, Merck). The residues were hydrolyzed as reported previously[23],[24]. In addition, ^1^H-NMR spectrum was taken. The prediction of toxicity and various properties like chemical, physicochemical, structural, quantum/classical mechanics were performed on the PreADME server.

### Theoretical model

To explain the NMR spectra, firstly, the character of a spin-active nucleus (like proton) of pesticides was investigated. The precise frequencies at which the spin active nuclei resonated were picked-up and displayed in the spectra. There were two types of orientations, α and β spin states, which differed very slightly in energy. This energy difference was supplied by the radiofrequency radiation to allow the nuclear spins to change their states. The energy difference (▵*E*) between spin states was directly proportional to the magnetic field strength. Based on this assumption and the Planck's law: 
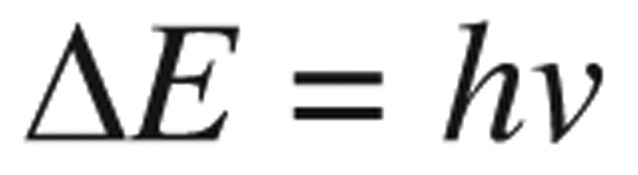
(1) Where *h* was Planck's constant and *v* was a frequency of electromagnetic radiation (EM)-radiation. 
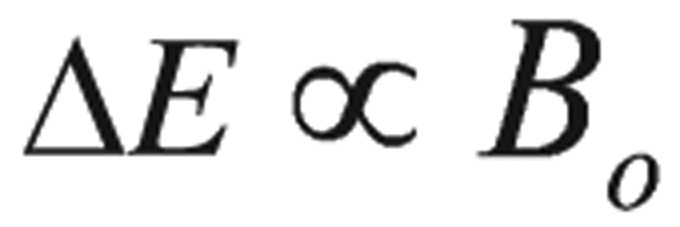
(2) Or 
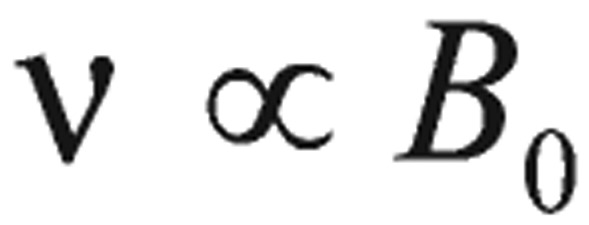
(3)

Larmor frequency (*v*) was directly proportional to the strength of the external magnetic field and 
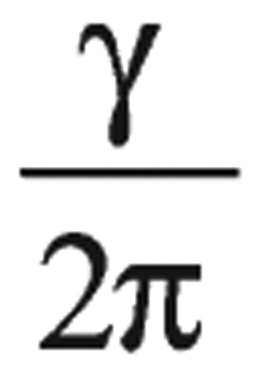
 was the proportionality constant, where γ was the magnetogyric ratio of the nucleus, which was the proportionality constant between magnetic moment µ and the spin number I: 
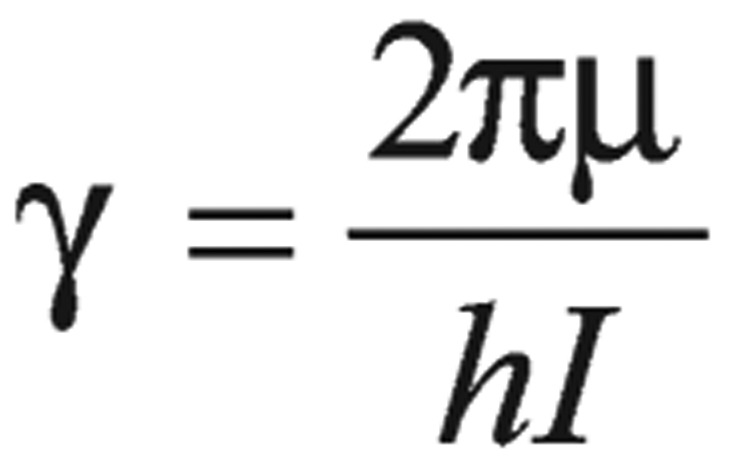
(4)

A pure sample of unknown was used as the method for standard additions. In this method, two samples were prepared. One was unknown. Another was the mixture of known volume of the unknown and a standard solution prepared from the pure pesticides. The integrated resonance of the unknown (*A*_x_) and the mixture (*A*_m_) were calculated as follows: 
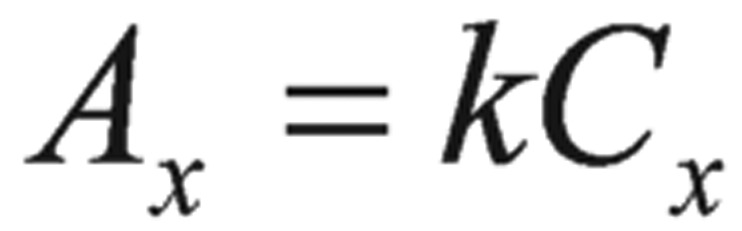
(5)

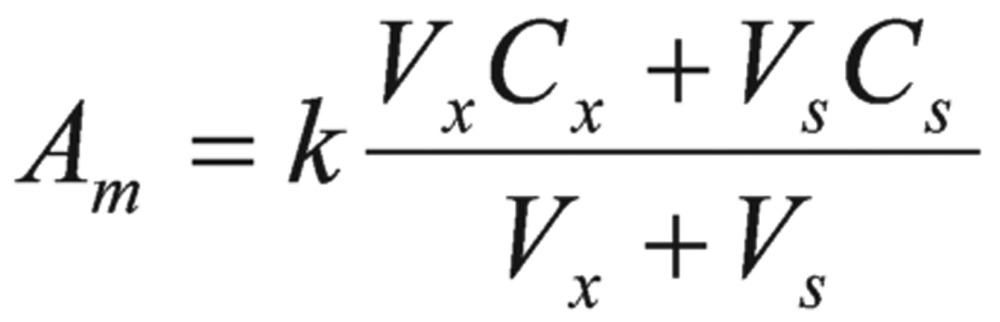
(6)

Where k was the proportionality constant, Cx and Cs were concentrations of the unknown and standard, respectively, Vx and Vs were the volumes of the unknown and standard in the mixture, respectively. 
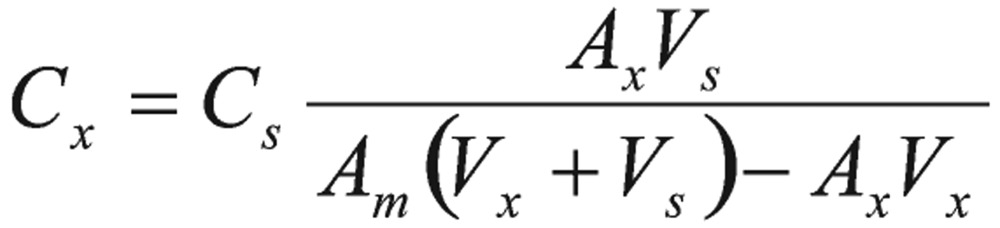
(7)

The advantages to this method were that fewer samples were needed to be prepared than for the calibration curve method, and it also had a tendency to compensate for any unsuspected chemical effects. Therefore, the concentration of the unknown may be calculated as follows: 
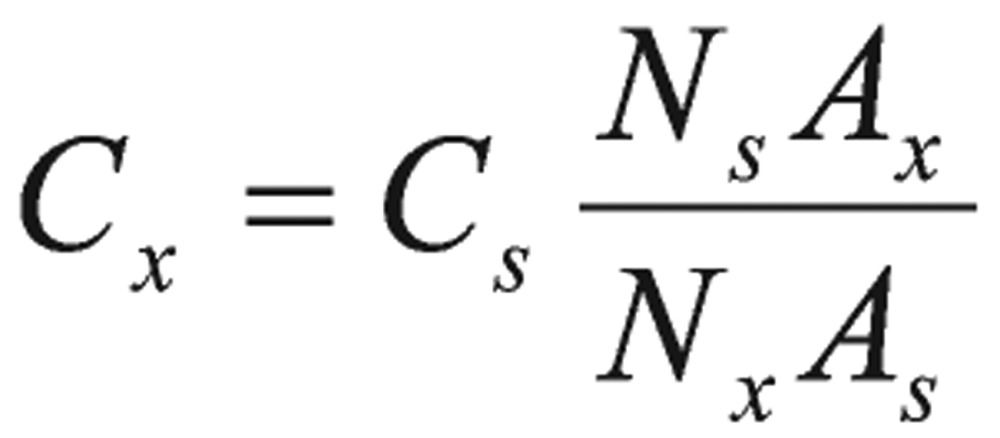
(8)

Where *C_x_* and *C_s_* were concentrations (mole units) of the unknown and standard toxic pesticide samples, respectively, *N_x_* and *N_s_* were the number of protons, and *A_x_* and *A_s_* were the corresponding integrated areas. 
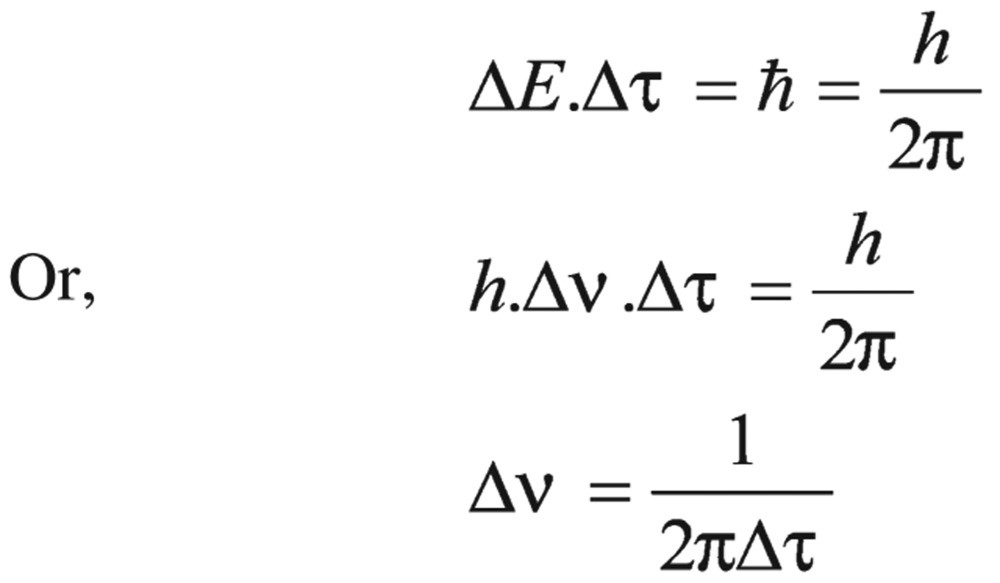
(9)

▵*v* value was an approximation of the line width and ▵τ may be expressed as the mean lifetime of the nucleus under a given magnetic environment.

The magnitudes of chemical shifts (*δ*) turned to be the order of parts of the operating frequency per million (10^−6^)[26]-[29]. 
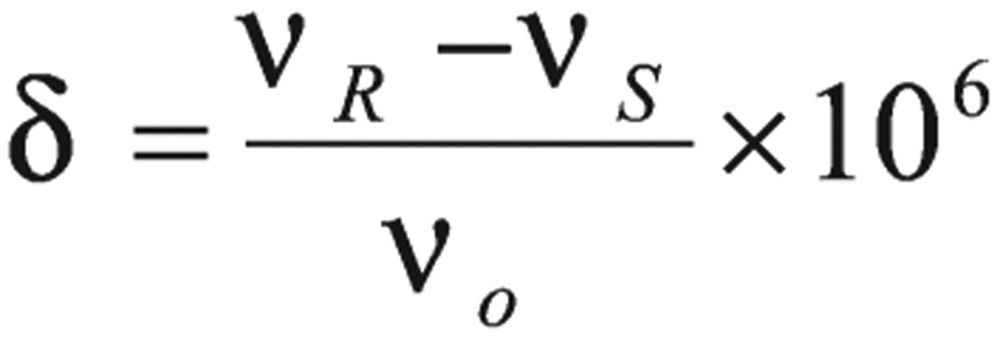
(10)

The splitting between two peaks of each doublet was the same and was said to be the coupling constant (*J*_ab_), where the subscript “ab” referred to coupled nuclei. It was independent of the size of external field or operating frequency. It meant that for a given coupled system, *J*_ab_ was the same whether the spectrum was recorded at 500 MHz or not.

## RESULTS

### The 3D structure of pesticides

**Fig. 1:**
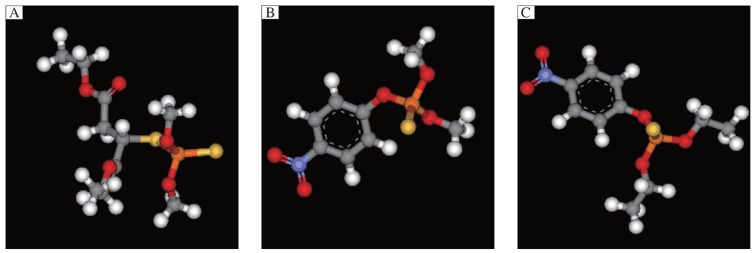
3D structures of malathion (A), parathion (B) and methyl parathion (C) using the RasMol visualization tool. The various elements in 3D structure of three pesticides contains hydrogen (white), oxygen (red), carbon (grey), sulphur (yellow), nitrogen (purple) and phosphorous (orange).

The 3D structures and the chemical, structural and physicochemical properties of the three pesticides were investigated based on the presence/absence of their functional chemical groups, which depended on the frequency of molecular atoms due to classical mechanics. This was especially true for minor changes in the molecular structure of some pollutants like methyl parathion, malathion and parathion, of which the structure possessed different phenomena compared with other compounds. The 3D structures of methyl parathion, malathion and parathion are shown in ***Fig. 1***. The 3D structures of the three pesticides were searched in the PubChem Compound Database. All the structures of the pesticides were downloaded from PubChem Compound Database and saved as *.sdf files, with the PubChem Id (CID) of 4004 for malathion, 4130 for methyl parathion, and 991 for CID, respectively. The coordinates of the pesticides were visualized using the RasMol V2.7.2.1.1 tool. The various elements in the 3D structures of the three pesticides (***Fig. 1***) contained hydrogen (white), oxygen (red), carbon (grey), sulfur (yellow), nitrogen (purple) and phosphorous (orange). The molecular formulas of the three pesticides were C_10_H_19_O_6_PS_2_ for malathion, C_10_H_14_NO_5_PS for parathion and C_8_H_10_NO_5_PS for methyl parathion.

### Prediction of toxicity

The toxicity of the pesticides was further predicted by using the PreADME server tool (http://www.bmdrc.org/04_product/01_preadme.asp), which computes and validates the toxic and environmental effects of chemicals solely from their molecular structures. ADMET_BBB predicted blood-brain barrier (BBB) penetration after oral administration of any lead molecules in the human body. Malathion, parathion and methyl parathion all showed low penetration in the BBB in the human body (***Table 1***).

**Table 1:** PreADME descriptors chart for toxic pesticides

3D structure of pesticides	Analysed computational properties of pesticides	Malathion 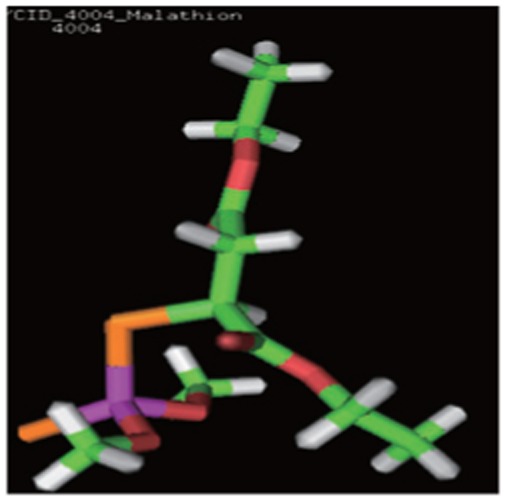	Parathion 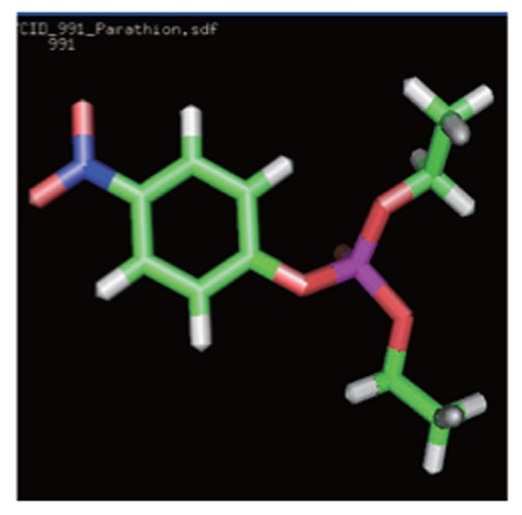	Methyl parathion 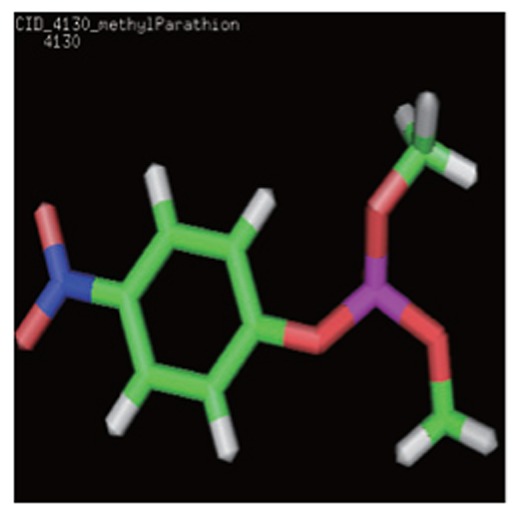
Ligand 3-D Structure: The 3-D structures of all the three pesticides contain various atoms like hydrogen (white), oxygen (red), carbon (green), sulfur (orange), nitrogen (blue) and phosphorous (pink).	ADMET_BBB	-0.774	-0.396	-0.18
	ADMET_BBB _Level	3	2	2
	ADMET_S olubility	-2.093	-3.585	-4.091
	ADMET_S olubility _Level	3	3	2
	ADMET_Hepatotoxicity	0	1	1
	ADMET_Hepatotoxicity_Probability	0.139	0.629	0.523
	ADMET_CYP2D6_Probability	0.247	0.029	0.108
	ADMET_PPB_Level	0	0	0
	ADMET_AlogP98	1.595	2.524	3.221
	ADMET_PSA_2D	70.321	64.595	64.595
				
Molecular Descriptors (2D Constitutional Descriptors)	Number of total atoms	38	32	26
	Number of H atoms	19	14	10
	Number of C atoms	10	10	8
	Number of N atoms	0	1	1
	Number of O atoms	6	5	5
	Number of P atoms	1	1	1
	Number of S atoms	2	1	1
	Fraction of hetero atoms	0.236842	0.250000	0.307692
	Molecular weight	330.350260	291.258060	263.204460
	Molecular formula	C10H19O6PS2	C10H14NO5PS	C8H10NO5PS
	Number of rigid bonds	37	32	26
	Number of total bonds	37	32	26
	Number of single bonds	34	24	18
	Number of double bonds	3	2	2
	Number of C-C bonds	5	2	0
	Number of C-O bonds	6	3	3
	Number of C=O bonds	2	0	0
	Number of C-S bonds	1	0	0
	Number of C-H bonds	19	14	10
	Number of P-O bonds	2	3	3
				
Chemical Feature Count	Number of H-bond acceptors	6	5	5
				
Functional Group Count	Number of alcohol groups	2	0	0
	Number of ether groups	2	0	0
	Number of ester groups	2	0	0
	Number of 6 member rings	0	1	1
				
2D Electrostatic Descriptors (Charged Partial Surface Area Descriptors i.e. Jurs Descriptors)	PPSA1 (partial positive surface area 1st type)	326.987478	147.579800	113.456719
	PPSA2 (partial positive surface area 2nd type)	578.937713	169.508516	127.121870
	PPSA3 (partial positive surface area 3rd type)	20.181663	9.485652	9.418458
	PNSA1 (partial negative surface area 1st type)	146.492709	212.513082	195.110454
	PNSA2 (partial negative surface area 2nd type)	-632.009335	-898.187603	-819.257510
	PNSA3 (partial negative surface area 2nd type	-43.308893	-38.095599	-40.074718
	Hydrophobic surface area (MPEOE method)	313.846235	284.087153	232.561444
	Positive charged polar surface area (MPEOE method)	46.149730	19.618244	19.618244
	Negative charged polar surface area (MPEOE method)	113.484223	56.387485	56.387485
	HRNCS (relative negative charged surface area to H-bond acceptors atoms)	15.189368	16.130962	16.125670
				
Partial Charge of Atom	Q_max_ (Maximum partial charge)	0.342842	0.344081	0.343241
	Q_min_ (Minimum partial charge)	-0.634495	-0.875633	-0.876345
	Total positive charge	1.770520	1.148589	1.120444
	Total negative charge	-4.314272	-4.226505	-4.198942
	Charge polarization	0.160126	0.167972	0.204592
				
2D Geometrical Descriptors	Hydrophobic surface area	274.628332	224.731649	190.267480
	Hydrophobic surface area (saturated group)	229.152497	127.113337	92.649168
	Hydrophobic surface area (unsaturated group)	45.475835	97.618312	97.618312
	Other group surface area	29.416996	15.158069	15.158069
	Polar surface area	41.473302	51.957001	51.957001
	H-bond acceptor surface area	41.473302	47.928966	47.928966
	H-bond donor surface area	0.000000	0.00000	0.000000
	H-bond surface area	41.473302	47.928966	47.928966
2D Physicochemical Descriptors	Polarizability (Miller method)	30.498000	26.530000	22.860000
	SK logP (SK atomic types)	2.225320	3.625450	2.882770
	Water solubility in pure water (SK atomic types, mg/L)	582.391	25.0569	63.4692
	Water solubility in buffer system (SK atomic types, mg/L)	10.503	0.5291	2.08965
	SK logVP (SK atomic types, log vapour pressure)	-5.656338	-4.398758	-4.104238
	Water solvation free energy (Ghose method)	-3.460000	0.150000	-0.510000
	AlogP98 (Ghose method)	2.160900	3.275900	2.578300
2D Topological Descriptors	S_hetero atoms	40.613179	35.572852	34.510740
	S_hydrophobic	3.240905	7.782440	5.967146
	S_polar	44.773520	38.719081	37.563251
	S_H bond acceptor	44.773520	40.162152	39.012001

The slightly toxic pesticides had a lower molecular weight in comparison with the most toxic pesticide, suggesting that the molecular weight can be used to assess the toxicity level of a pesticide. The investigated groups of the pesticides differed in their molecular weights accordingly. The hydrogen atoms present in pesticides carry a relatively electronegative atom and positive charge to become more reactive. Therefore, they act as hydrogen bond donors in the formation of a hydrogen bond with electronegative atoms such as oxygen or phosphorus and sulfur that function as hydrogen bond acceptors. These donors and acceptors are ideal components of toxic pesticides due to their high reactivity. The lowest number of hydrogen bond acceptors complied with the Lipinski's rules[29]. According to this rule, it is supposed that no more than 10 hydrogen bond acceptors are detected in compounds with ADME properties[30].

The distribution of functional groups in toxic pesticides is presented in ***Table 1***. The results showed that the occurrences of functional groups increased with the toxicity. Hydroxyl groups made molecules more reactive, which is an important property for toxic pesticides during the hydrolysis process. Analysis of the structural characteristics provides the information regarding functional properties. It has been indicated that the center of aromatic rings acts as hydrogen acceptors and plays a significant role in molecular associations[31]. The concept of interactions between pesticides with metabolic proteins offers knowledge about bond strength, quantum mechanical charge distribution and organic reaction mechanisms.

Absorption depends on the solubility and permeability of the pesticide as well as interactions with transporters and metabolic enzymes. The consideration during this stage ensures the solubility and lipophilicity (i.e. hydrophobicity) for optimal absorption[32],[33]. The relationship between important ADME parameters and molecular structural properties was discussed in *in-silico* models to predict the ADMET properties[34]. The ADME properties focus on intestinal permeability, solubility, human intestinal absorption, BBB permeation, plasma protein binding and metabolic stability. The distribution of pesticides depends on their structural and physicochemical properties. Firstly, when pesticide binds with its molecular target, if the affinity of the pesticide is too high for the target, the pesticide molecule follows the law of mass action and the pesticide molecule does not bind to the target after eliciting its response. Secondly, other molecules of the pesticides reach the particular sites with plasma proteins[35]. The computational prediction of BBB permeation has been done previously in three steps. In the first step, there are simple “rules of thumb” which are derived by examining the molecule properties of pesticides crossing the BBB or not. The second step of BBB is to predict whether a pesticide is a BBB permeator or not. The third step defines the logarithmic BBB permeation analysis. Another factor of PreADME is clearance or excretion, which shows the frequency of pesticide in BBB. The highly polar pesticides are lipid soluble and reabsorbed from the blood stream. In addition, they enter into metabolism and generate more polar species.

In the retention time, pesticides are eliminated from their metabolic environments. Good affinity may be not necessary if the retention time is too long, which causes toxic effects. On the other hand, long retention time could be potentially advantageous in terms of binding effect on the basis of dissociation constants.

### ^1^H-NMR spectral analysis

#### Malathion

**Fig. 2:**
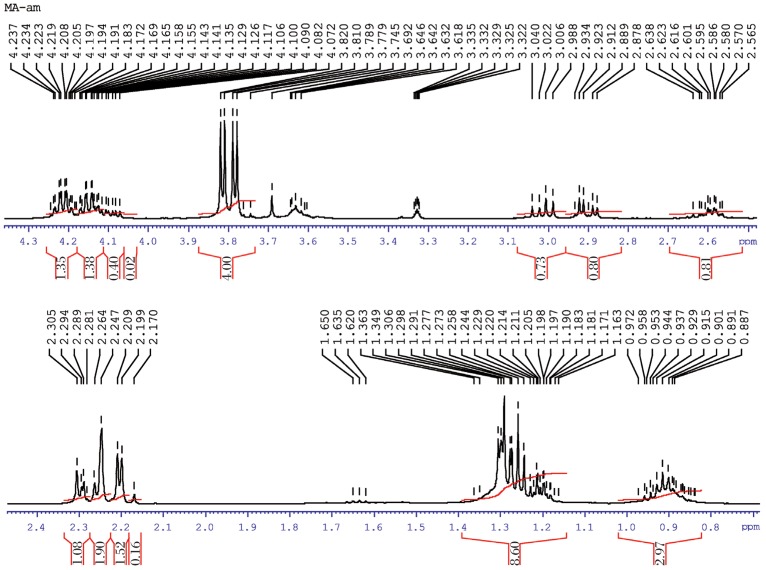
^1^H-NMR spectra of pure malathion (1 ppm) for analysis of hydrolyzed products.

***Fig. 2*** shows the ^1^H-NMR spectra of pure O, O-dimethyl-5-(1, 2-dicarbethoxyethyl) phosphorodithionate (malathion) in CdCl_3_ solvent. The methyl quartets a and a′ were readily seen in two overlapping triplets. The methylene protons at d and d′ confirmed two closely spaced triplets centered at about 4.2 ppm by the proton decoupling at the methyl resonance frequency. The resulting singlets for the d and d′ protons are shown in molecular structure of malathion. The methylene protons at b and b′ were nonequivalent for symmetry. In addition, these protons were spincoupled with the methine proton at e and therefore, the resonances of the b and b′ protons appeared as the AB part of an ABX pattern. The protons at e gave resonance lines that lay beneath the pattern of the d and d′ protons, as explained in the following structure. 
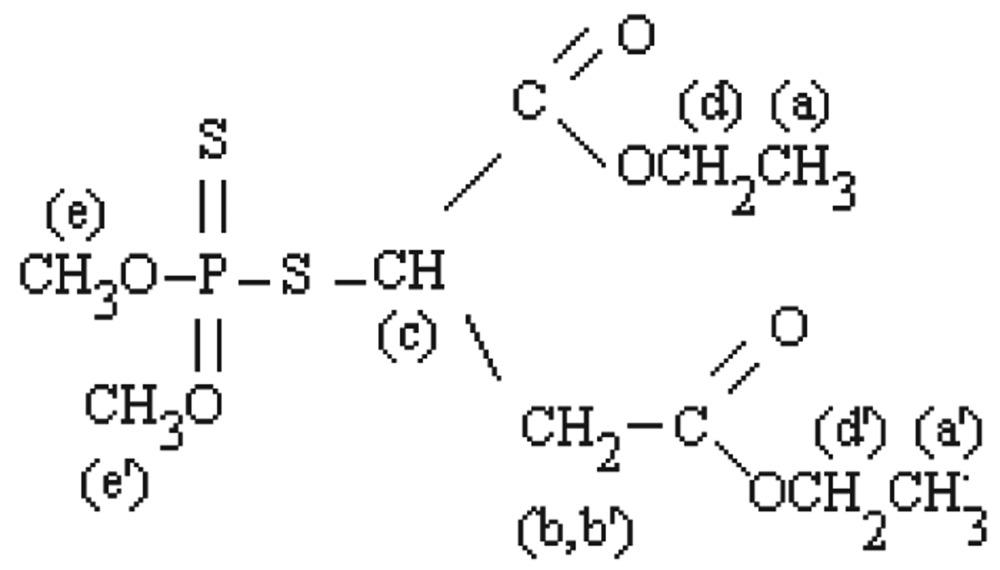


#### Malathion hydrolysis


**Fig. 3:**
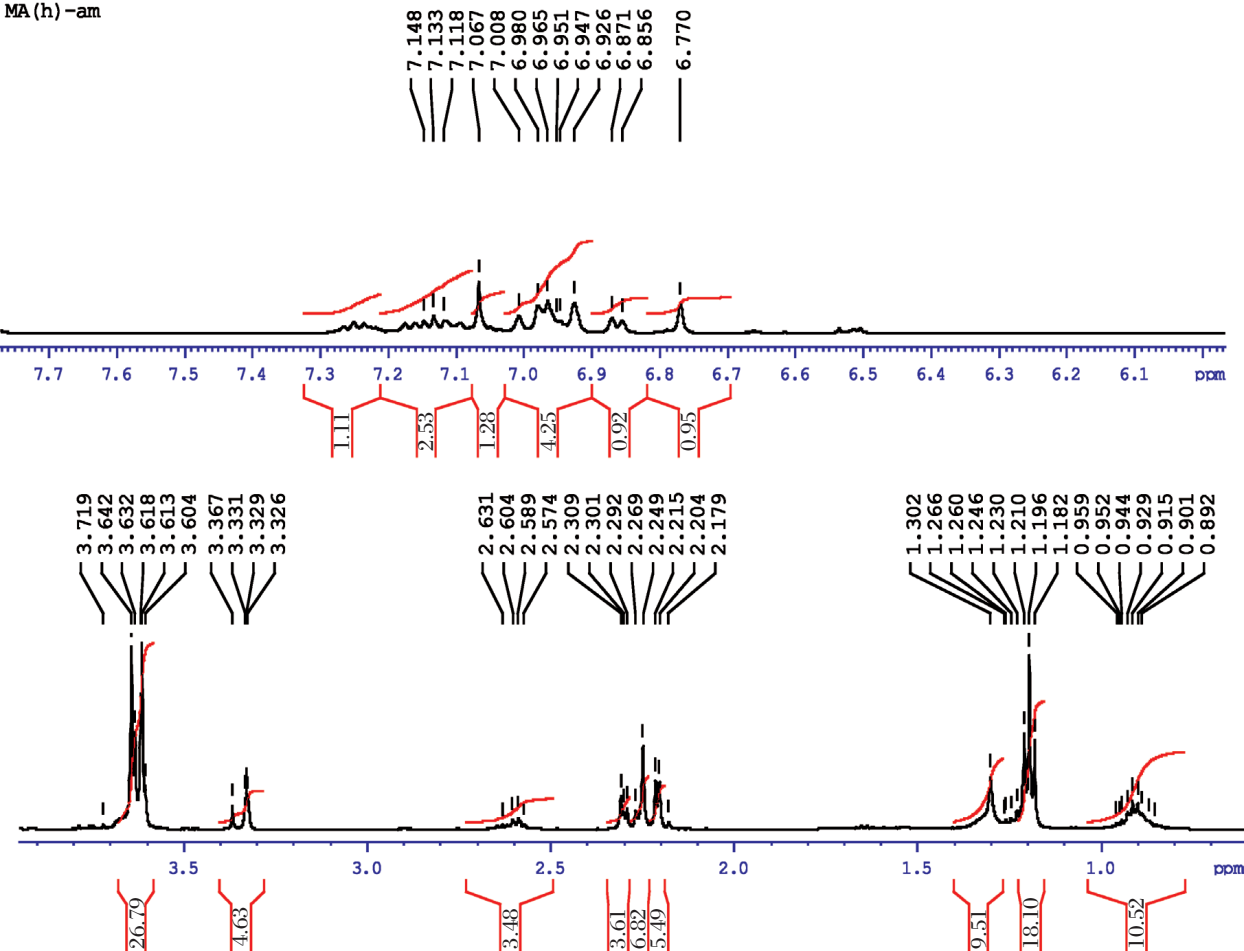
^1^H-NMR spectra of hydrolyzed malathion (1 ppm) sample.

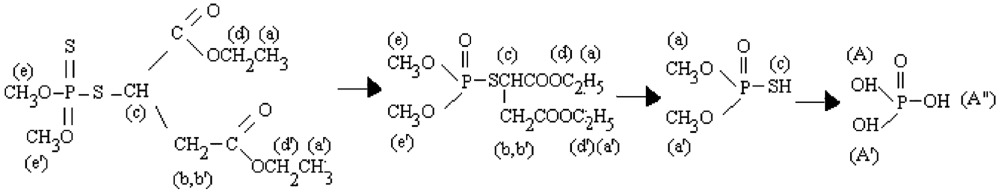
 During hydrolysis of malathion, the methyl quartets a and a′ presented two overlapping triplets (***Fig. 3***). The methylene protons at d and d′ confirmed two closely spaced triplets centered at about 4.2 ppm by the proton decoupling at the methyl resonance frequency. The resultant singlets for the d and d′ protons were shown in the molecular structure of malathion. The methylene protons at b and b′ were nonequivalent for symmetry due to the production of oxidative products of malathion (malaoxon). In addition, these protons were spin-coupled with methine protons at e and therefore, the resonance of the b and b′ protons appeared in an ABX pattern. The protons at A, A′ and A′ gave resonance lines that lay beneath the pattern of the e and e′ protons. The ^1^H-NMR results of malathion and malathion hydrolysis are presented in ***Table 2***.

**Table 2:** ^1^H-NMR spectral characterization of malathion and hydrolyzed malathion

H-atom position in the MA structure	Malathion (MA)					H-atom positionin the MA(H)structure	Malathion [MA(H)]			
	Chemical shift (ppm)		Coupling constant (J in Hz)	Integration	Relaxation time (sec)		Chemical shift (ppm)	Coupling constant (J in Hz)	Integration	Relaxation time (sec)
aa′ (6H)	Double		10.0	1:10	0.2	aa′ (OH)	-----	-----	-----	-----
	doublet,					bb′ (2H)	Double	5.0-8.0	1:1	0.15
	4.205, 4.208,						doublet,			
	4.219, 4.223						2.631, 2.604,			
							2.589, 2.574			
bb′ (2H)	Double		5.0-8.0	1:3	0.15					
	doublet,					c (1H)	Singlet, 6.770	490-650	1:3	0.16
	2.98, 3.0,									
	3.02, 3.04									
c (1H)	Singlet, 4.1		4.0-6.0	1:5	0.16	dd′ (OH)	-----	-----	-----	-----
dd′ (4H)	Doublet,		0.8	1:3	0.25	ee′ (6H)	Doublet,	9.0-10.0	1:1	0.11
	2.87, 2.88						3.719, 3.642			
ee′ (6H)	Doublet, 3.81, 3.82		9.0-10.0	1:4	0.11	A(1H)	7.148	490-650	1:3	0.02
						A′ (1H)	7.133	490-650	1:3	0.02
						A′ (1H)	7.118	490-650	1:3	0.02

#### Methyl parathion

**Fig. 4:**
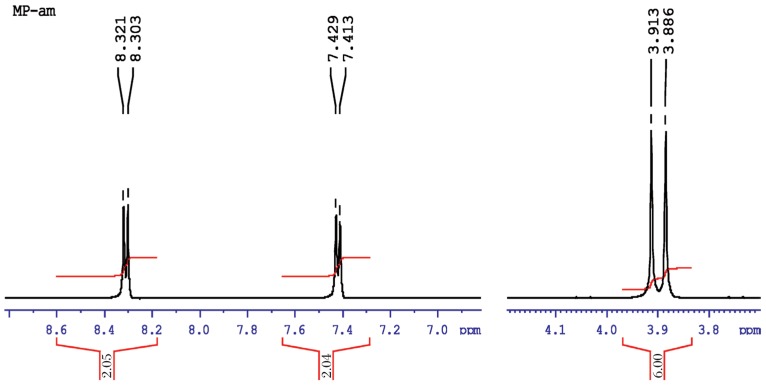
^1^H-NMR spectra of pure methyl parathion (1 ppm) for for analysis of hydrolyzed products.

The molecular structure of O, O-dimethyl O-4-nitrophenylhiosphorothioate is shown as follows: 
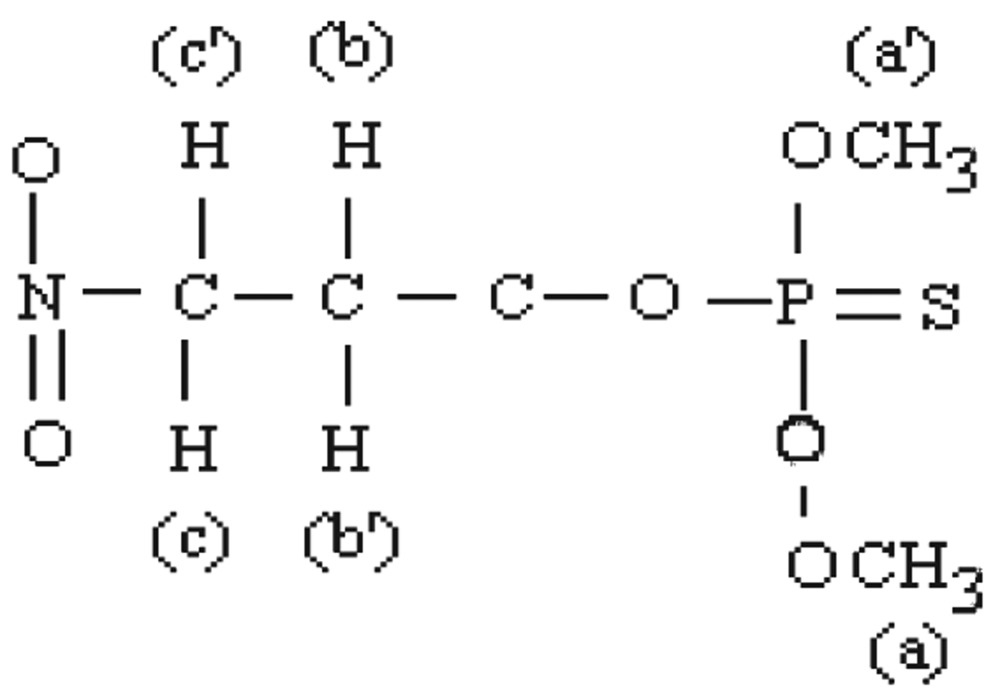


***Fig. 4*** shows the NMR spectra of methyl parathion. The methyl quartets a and a′ indicated two overlapping triplets. The methylene protons at bb′ and cc′ confirmed two closely spaced triplets centered at about 7.4-8.3 ppm by the proton decoupling at the methyl resonance frequency. The methylene protons at b and b′ (doublet) and at c and c′ (doublet) were nonequivalent for symmetry. Therefore, the resonance of the doublets protons appeared as the AB part of an ABX pattern.

#### Methyl parathion hydrolysis

**Fig. 5:**
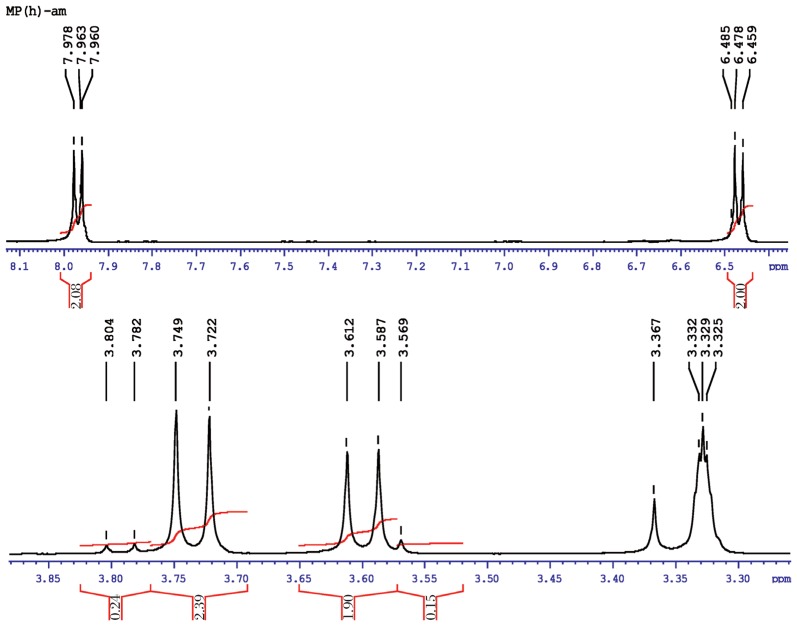
^1^H-NMR spectra of hydrolyzed methyl parathion (1 ppm) sample.

The molecular structure of hydrolyzed product of O, O-dimethyl O-4-nitrophenylhiosphorothioate may be presented as follows: 
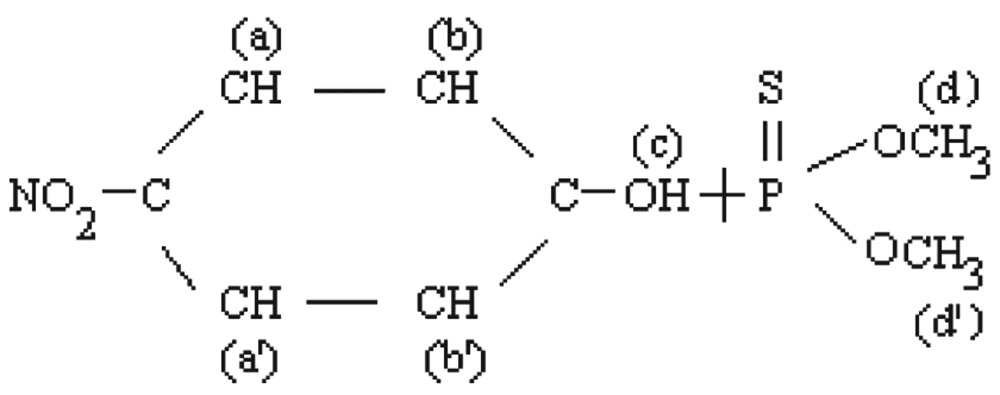


***Fig. 5*** shows the NMR spectra of methyl parathion hydrolyzed products. The ^1^H-NMR results of methyl parathion and methyl parathion hydrolysis are shown in ***Table 3***. The methyl quartets d and d′ indicated two overlapping triplets. The methylene protons at a and a′ confirmed two closely spaced triplets centered at about 3.7 ppm by the proton decoupling at the methyl resonance frequency. The methylene protons at b and b′ (doublet), and at c (singlet) were nonequivalent for symmetry. Therefore, the resonance of the doublet protons appeared as the AB part of an ABX pattern.

**Table 3:** ^1^H-NMR spectral characterization of methyl parathion and hydrolyzed methyl parathion

H-atom positionin the MP structure	Methyl Parathion (MP)				H-atom positionin the MP(H)structure	Methyl parathion [MP(H)]			
	Chemical shift (ppm)	Coupling constant (J in Hz)	Integration	Relaxation time (sec)		Chemical shift (ppm)	Coupling constant (J in Hz)	Integration	Relaxation time (sec)
aa′ (6H)	Singlet, 3.913	11.0-16.0	1:3	0.11	dd′(6H)	Double triplets, 7.978, 7.963, 7.960, 6.485, 6.478, 6.459	11.0-16.0	1:8	0.11
bb′ (2H)	Doublet, 7.429, 7.413	1.0-3.0	1:1	0.5					
cc′ (2H)	Doublet, 8.321, 8.303	1.0-3.0	1:1	0.5	c(lH)	Singlet, 3.569	1.0-3.0	2:1	0.5
					aa′ (2H)	Doublet, 3.749, 3.722	1.0-3.0	1:2	0.5
					bb′ (2H)	Doublet, 3.612, 3.587	1.0-3.0	1:8	0.16

#### Parathion

**Fig. 6:**
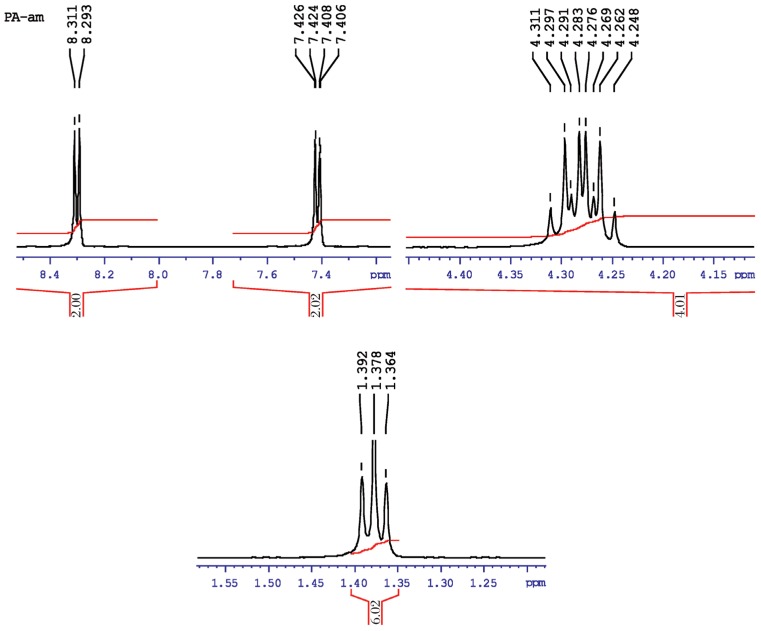
^1^H-NMR spectra of pure parathion (1 ppm) for analysis of hydrolyzed products.

***Fig. 6*** shows the NMR spectra O, O-diethyl O-4-nitrophenylphosphorothioate (also known as parathion), which is often represented in its simplest form as: 
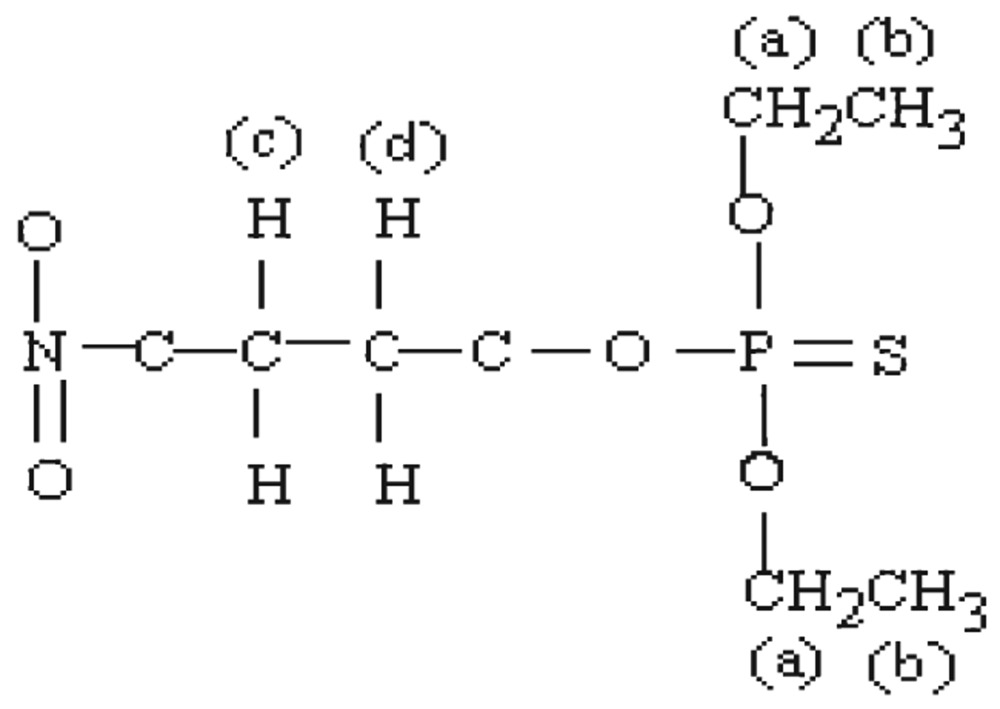


It represented the methyl doublet at a and a′, and b and b′ (symmetry) represented the singlet at 4.2 ppm. The methylene protons at d and d′, and c and c′ confirmed two closely spaced doublets centered at about 7.4-8.4 ppm by the proton decoupling at the methyl resonance frequency.

#### Parathion hydrolysis

**Fig. 7:**
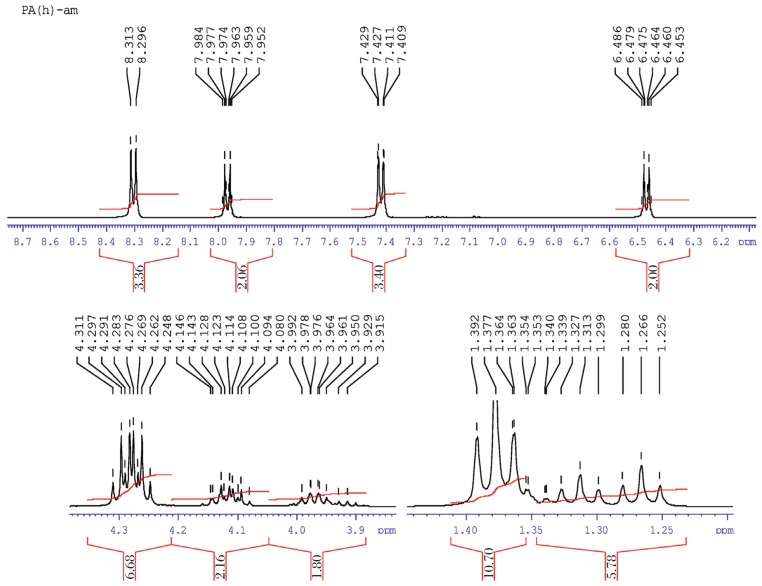
^1^H-NMR spectra of hydrolyzed (1 ppm) parathion sample.

It represented the methyl doublet at e and e′, which indicated the singlet proton at 1.3 ppm due to the symmetry and at d and d′ that revealed the doublet at 4.3 ppm. The methylene protons at a and a′, and at b and b′ confirmed that two closely spaced doublets present were centered at about 7.4-8.3 ppm by the proton decoupling at the methyl resonance frequency coupled at singlet c (3.978 ppm). The ^1^H-NMR results of parathion and parathion hydrolysis are shown in ***Table 4***. In ***Fig. 7***, the NMR spectra of O, O-diethyl O-4-nitrophenylphosphorothioate and the hydrolyzed products can be represented in molecular form as: 
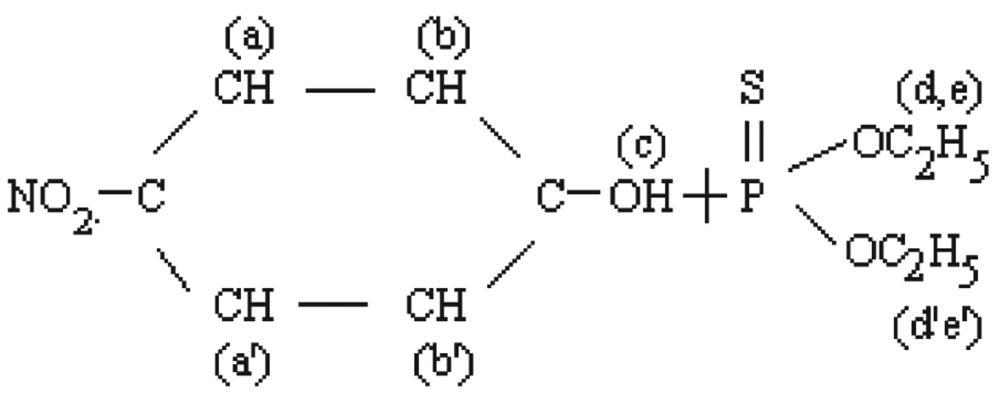


**Table 4:** ^1^H-NMR spectral characterization of parathion and hydrolyzed parathion

H-atom positionin the PA structure	Parathion (PA)				H-atom positioiin the PA(H)structure	Parathion [PA(H)]			
	Chemical shift (ppm)	Coupling constant (J in Hz)	Integration	Relaxation time (sec)		Chemical shift (ppm)	Coupling constant (J in Hz)	Integration	Relaxation time (sec)
aa′ (6H)	Singlet, 1.392	4.0-6.0	1:3	0.2	aa′ bb′ (4H)	Double doublet, 7.952, 7.429,	1.0-3.0	1:2	0.5
bb′ (4H)	Doublet, 4.297, 4.291	8.0-10.0	1:2	0.05					
					c (1H)	8.313, 8.296	1.0-3.0	1:1	0.5
cc′ (2H)	Doublet, 8.311, 8.293	6.0-8.0	1:1	0.05	dd′ (4H)	Singlet, 3.978	4.0-6.0	1:1	0.2
					ee′ (6H)	Doublet,	8.0-10.0	1:6	0.11
dd′ (2H)	Doublet, 7.426, 7.424	9.0-10.0	1:1	0.07		4.311, 4.297 Singlet, 1.392			

### Quantification of toxic pesticides

**Fig. 8:**
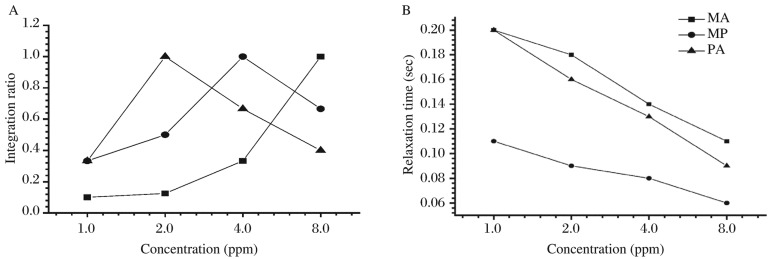
A quantitative plot between concentration (ppm) and proton integration ratio (A) and relaxation time (s) (B) of different pesticides. MA: malathion; MP: Methyl parathion; PA: Parathion.

In this study, two parameters (integration and relaxation time) were used to determine the quantity of toxic pesticides in comparison to the pure spectra. The present method was used for qualitative and quantitative analyses of toxic pesticides. The resultant data can be used to predict the toxicity of pesticides under different environments. ***Fig. 8*** demonstrated the plot between concentration and integration, concentration and relaxation time. All samples were prepared freshly to ensure accuracy. The quantification of toxic pesticides is presented in ***Table 5*** calculated using the aforementioned equations 1, 7 and 10[25]-[28]. The results showed that the integration increased with the concentration of pesticides, while the relaxation time decreased with the increased concentration of toxic pesticides (***Fig. 8***). Therefore, the present data can be utilized to explore the presence of these compounds in the complex form.

**Table 5:** ^1^H-NMR spectral characterization of pesticides at different concentration.

Malathion (MA)			Methyl parathion (MP)			Parathion (PA)		
Concentration	Integration	Relaxation time(s)	Concentration	Integration	Relaxation time(s)	Concentration	Integration	Relaxation time(s)
1.0 ppm	1:10	0.2	1.0 ppm	1:3	0.11	1.0 ppm	1:3	0.2
2.0 ppm	1:8	0.18	2.0 ppm	1:2	0.09	2.0 ppm	1:1	0.16
4.0 ppm	1:3	0.14	4.0 ppm	2:1	0.08	4.0 ppm	2:3	0.13
8.0 ppm	1:1	0.11	8.0 ppm	2:3	0.06	8.0 ppm	2:5	0.09

## DISCUSSION

The ADMET_Absorption_Level in the lead compounds has good absorption level in human intestinal absorption (HIA) after oral administration. The solubility of malathion was -2.093, and -3.585 and -4.091 for parathion and methyl parathion in the aqueous media (***Table 1***). The ADMET_Hepatotoxicity model can be used to predict potential organ toxicity for a wide range of structurally diverse compounds. The results showed that malathion was not toxic while the other two pesticides were toxic in nature. ADMET_Hepatotoxicity_Probability is the hepatotoxicity score, which is the sum of the predicted values (0 and 1) from all individual trees that comprise the ensemble recursive partitioning model divided by the total number of trees in the model. Parathion and methyl parathion were similarly toxic while malathion was non-toxic because the value was approximate to 0.

The present study focused on the detection procedure of ^1^H-NMR based on hydrolysis. This method uses a radiofrequency excitation and records the spectrum of the non-hydrolyzed and hydrolyzed toxic pesticide samples. The ^1^H-NMR spectra of toxic pesticides such as malathion, methyl parathion and parathion are shown in ***Fig. 2*** to ***Fig. 7***, and their parameters are calculated in ***Table 2*** and ***Table 4***. In this method, the active groups with ^1^H-atoms in toxic organophosphate pesticides like ester, nitro or phenolic group in malathion, methyl parathion and parathion were found in which light-to-dark-yellow was present with the addition of slight amount of sodium hydroxide and hydroxyl ammonium hydrochloride, which was attributed to the breakage of the spin active proton groups in the compounds. Such a cause is valid on the basis of previously reported studies[3],[14],[24],[25]. The quantification procedure was formed by estimation of toxic pesticides using the ^1^H-NMR results, which are presented in ***Fig. 8*** and the data are summarized in ***Table 5***. The spin-active data can be utilized through quantitative and qualitative analyses of toxic pesticides under different environments. The variation in integration and relaxation time for malathion, methyl parathion and parathion is due to the various structures of different chemicals. However, the experimental procedure was the same for all. The different relaxation time for malathion, methyl parathion and parathion was closely related to the orientations of different pesticides. The lower relaxation time is due to low affinity and shows less stability, the higher relaxation time shows increasing toxicity level. Therefore, the relaxation time is considered as an indicator for predicting the toxicity of a compound. The mechanism of predicting toxic pesticides showed a high response to the reaction as well as ^1^H-NMR characteristics. The results of qualitative and quantitative analyses of these pesticides revealed that the amounts of malathion, methyl parathion and parathion were determined using the ^1^H-NMR data. The values of four parameters such as chemical shift, coupling constant, integration and relaxation time were correlated with the concentration of toxic pesticides, and they can be used to characterize the proton groups in the molecular structures of toxic pesticides. All data are reproducible under certain environment conditions and can be applied for quantitative analysis of toxic pesticides under different environments.

The present study investigated the structural, chemical and functional properties of pesticides. The analyses of hydrogen bond donors and acceptors with structural features showed a positive correlation between occurrence and toxicity, whereas the amount of pesticides is closely related to ^1^H-NMR analysis. The combination of basic analyses in chemical bonding, quantum mechanics (i.e. charge distribution and geometric analysis), classical mechanics (i.e. molecular mechanics), thermodynamics (i.e. free energy of complex formation) and statistical mechanics (i.e. configuration) was presented in a tabulated form. The significance of four ^1^H-NMR parameters is well interfaced with the discussion of PreADME. Furthermore, NMR has been routinely used to predict the qualitative and quantitative analyses of toxic pesticides and other bioactive chemical compounds. The use of high-resolution NMR for the elucidation of structure of pesticides is also characterized.

## References

[1] Sharma AK, Gaur K, Tiwari RK, Gaur MS (2011). Computational interaction analysis of organophosphorous pesticides with different metabolic protein in human. J Biomed Res.

[2] Koo IS, Ali D, Yang K, Park Y, Yong AE, Vanloon GW (2009). 31P NMR and ESI-MS studies of metal ion-phosphorus pesticide residue complexes. Can J Chem.

[3] Tamura H, Yoshikawa H, Gaido KW, Ross SM, De-Lisle RK, Welsh WJ (2003). Interaction of organophosphate pesticides and related compounds with the androgen receptor. Environ Health Perspect.

[4] Sanghi R (2003). Organochlorine and organophosphorus pesticide residues in breast milk from Bhopal, Madhya Pradesh, India. Hum Exp Toxicol.

[5] Doreen C, Jason CFC, Yong PS, Smith VH, Gary V, Buncel E (2006). Complexation of diazinon, an organophosphorus pesticide, with α-, β-, and γ-cyclodextrin-NMR and computational studies. Can J Chem.

[6] Yuk J, Mc-Kelvie JR, Simpson MJ, Spraul M, Simpson AJ (2010). Comparison of 1-D and 2-D NMR techniques for screening earthworm responses to sub-lethal endosulfan exposure. Environ Chem.

[7] Yeasmin L, Mac-Dougall SA, Wagner BD (2009). UV-A photochemistry of the pesticide azinphos-methyl: Generation of the highly fluorescent intermediate N-methylanthranilic acid. J Photochem Photobiol A Chem.

[8] Descampiaux B, Imbenotte M, Desenclos V, Vermeersch G, Lhermitte M, Erb F (1997). 1H NMR investigation of toxic effects of lindane and paraquat on Hep 3B and Hep G2 human hepatoma cell lines. Chem Res Toxicol.

[9] Thomas A, Gerken W, Ritchey M (1969). Lanthanide-induced proton, carbon, and phosphorus NMR shifts for a series of organophosphorus compounds. J Magn Reson.

[10] Rackham David M (1976). Recent applications of quantitative nuclear magnetic resonance spectroscopy in pharmaceutical research. Talanta.

[11] Donald EL, Jerry FW (1968). Nuclear magnetic resonance line widths and linear extrapolation chelometric titrations. Anal Lett.

[12] Gerald D (1975). Computerized signal processing. Anal Chem.

[13] Arnold JT (1956). Nuclear magnetic resonance spectra of some hydrocarbons. Phys Rev.

[14] Cohn M, Leigh JS (1962). Magnetic resonance investigations of ternary complexes of enzyme-metal-substrate. Nature.

[15] Lavertu M, Xia Z, Serreqi AN, Berrada M, Rodrigues A, Wang D (2003). A validated 1H NMR method for the determination of the degree of deacetylation of chitosan. J Pharm Biomed Anal.

[16] Koo IS, Ali D, Yang K, Park Y, Wardlaw DM, Buncel E (2008). Theoretical study of 31P NMR chemical shifts for organophosphorus esters, their anions and O,O-dimethylthiophosphorate anion with metal complexes. Bull Korean Chem Soc.

[17] Becke AD (1993). Density functional thermo chemistry. III. The role of exact exchange. J Chem Phys.

[18] Ruiz-Morales Y, Ziegler T, Yosadara Ruiz-M, Tom Ziegler A (1998). Theoretical study of 31P and 95Mo NMR chemical shifts in M (CO) 5PR3 (M = Cr, Mo; R = H, CH3, C6H5, F, and Cl) based on density functional theory and gauge-including atomic orbitals. J Phys Chem A.

[19] Schreckenbach G, Tom Z (1995). Calculation of NMR shielding tensors using gauge-including atomic orbitals and modern density functional theory. J Phys Chem.

[20] Chesnut DB, Quin LD (2005). A study of NMR chemical shielding in 5-coordinate phosphorus compounds (phosphoranes). Tetrahedron.

[21] Schreckenbach G, Ziegler T (1997). Calculation of NMR shielding tensors based on density functional theory and a scalar relativistic pauli-type hamiltonian. The application to transition metal complexes. Int J Quant Chem.

[22] Balakrishnan VK, Julian MD, Van-Loon GW, Buncel E (2001). Catalytic pathways in the ethanolysis of fenitrothion, an organophosphorothioate pesticide: A dichotomy in the behaviour of crown/cryptand cation complexing agents. Can J Chem.

[23] Sharma AK, Gaur MS, Sharma P, Tiwari RK, Bhadoria S (2009). Development of colorimetric sensor instrument for quantitative analysis of methyl parathion. Sensor Rev.

[24] Gaur MS, Sharma AK, Sharma P, Mishra V, Tiwari RK (2008). Spectrophotometric assessments of methyl parathion in water samples. Can J Pure Appl Sci.

[25] Preston CM (1996). Applications of NMR to soil organic matter analysis: history and prospects. Soil Sci.

[26] Koskela H (2010). Use of NMR techniques for toxic organophosphorus compound profiling. J Chromatogr B Anal Technol Biomed Life Sci.

[27] Caligiani A, Acquotti D, Palla G, Bocchi V (2007). Identification and quantification of the main organic components of vinegars by high resolution 1H NMR spectroscopy. Anal Chim Acta.

[28] Belton PS, Delgadillo I, Holmes E, Nicholls A, Nicholson JK, Spraul M (1996). Use of high-field 1H NMR spectroscopy for the analysis of liquid foods. J Agric Food Chem.

[29] Lipinski CA, Lombardo F, Dominy BW, Feeney PJ (2001). Experimental and computational approaches to estimate solubility and permeability in drug discovery and development settings. Adv Drug Deliv Rev.

[30] Van de WH, Gifford E (2003). ADMET in silico modeling: towards prediction paradise?. Nat Rev Drug Discov.

[31] Levitt M, Perutz MF (1988). Aromatic rings act as hydrogen bond acceptors. J Mol Biol.

[32] Norinder U, Osterberg T (2001). Theoretical calculation and prediction of drug transport processes using simple parameters and partial least squares projections to latent structures (PLS) statistics. The use of electrotopological state indices. J Pharm Sci.

[33] Stenberg P, Norinder U, Luthman K, Artursson P (2001). Experimental and computational screening models for the prediction of intestinal drug absorption. J Med Chem.

[34] Schneider G, Fechner U (2005). Computer-based de novo design of drug like molecules. Nat Rev Drug Discov.

[35] Alavijeh MS, Chishty M, Qaiser MZ, Palmer AM (2005). Drug metabolism and pharmacokinetics, the blood-brain barrier and central nervous system drug discovery. NeuroRx.

